# Exploring the psychological impact of contact tracing work on staff during the COVID-19 pandemic

**DOI:** 10.1186/s12913-023-09566-6

**Published:** 2023-06-08

**Authors:** Hugh Fulham-McQuillan, Róisín O’Donovan, Claire M. Buckley, Philip Crowley, Brynne Gilmore, Jennifer Martin, Eilish McAuliffe, Gregory Martin, Gemma Moore, Mary Morrissey, Emma Nicholson, Éidín Ní Shé, Mary Clare O’Hara, Ricardo Segurado, Mary Rose Sweeney, Patrick Wall, Aoife De Brún

**Affiliations:** 1 UCD Centre for Interdisciplinary Research, Education, and Innovation in Health Systems (UCD IRIS), School of Nursing, Midwifery & Health Systems, University College Dublin, Dublin, Ireland; 2 Centre for Positive Psychology and Health, Royal College of Surgeons in Ireland (RCSI), Dublin 2, Ireland; 3 School of Public Health, University College Cork, Cork, Ireland; 4 Team Strategy and Research Directorate, Health Service Executive, Dublin, Ireland; 5 National Quality and Patient Safety Directorate, Health Service Executive, Dublin, Ireland; 6 Health Protection Surveillance Centre, Health Service Executive, Dublin, Ireland; 7 National Health Intelligence Unit, Research & Evidence, Health Service Executive, Dublin, Ireland; 8 Graduate School of Healthcare Management, Royal College of Surgeons in Ireland (RCSI), Dublin 2, Ireland; 9 Research and Development, Strategy and Research, Health Service Executive, Dublin, Ireland; 10 School of Public Health, Physiotherapy and Sports Science, University College Dublin, Dublin, Ireland; 11 School of Nursing, Psychotherapy and Community Health, Dublin City University, Dublin, Ireland

**Keywords:** Mental health, Psychological impact, Contact tracing staff, covid-19, Burnout, PTSD, Perceived stress, Mental distress, Health services research

## Abstract

**Background:**

Contact tracing is a key control measure in the response to the COVID-19 pandemic. While quantitative research has been conducted on the psychological impact of the pandemic on other frontline healthcare workers, none has explored the impact on contact tracing staff.

**Methods:**

A longitudinal study was conducted using two repeated measures with contact tracing staff employed in Ireland during the COVID-19 pandemic using two-tailed independent samples *t* tests and exploratory linear mixed models.

**Results:**

The study sample included 137 contact tracers in March 2021 (T1) and 218 in September 2021 (T3). There was an increase from T1 to T3 in burnout related exhaustion (p < 0·001), post-traumatic stress disorder (PTSD) symptom scores (p < 0·001), mental distress (p < 0·01), perceived stress (p < 0·001) and tension and pressure (p < 0·001). In those aged 18–30, there was an increase in exhaustion related burnout (p < 0·01), PTSD symptoms (p < 0·05), and tension and pressure scores (p < 0·05). Additionally, participants with a background in healthcare showed an increase in PTSD symptom scores by T3 (p < 0·001), reaching mean scores equivalent to those of participants who did not have a background in healthcare.

**Conclusions:**

Contact tracing staff working during the COVID-19 pandemic experienced an increase in adverse psychological outcomes. These findings highlight a need for further research on psychological supports required by contact tracing staff with differing demographic profiles.

**Supplementary Information:**

The online version contains supplementary material available at 10.1186/s12913-023-09566-6.

## Introduction

Contact tracing has been described by the World Health Organization (WHO) as a critical intervention to reduce transmission of the novel coronavirus (COVID-19) [1]. In response to the COVID-19 pandemic, the Irish Health Service Executive (HSE) rapidly implemented a large-scale contact tracing operation [2, 3]. After initially redeploying healthcare staff and public service workers, in addition to a volunteer workforce, to the role of contact tracing [4], the HSE recruited dedicated contact tracers, both clinically and non-clinically trained, from a variety of backgrounds, mainly through a third party recruiting agency [5]. As such, the current contact tracing workforce in Ireland is composed of professional health care workers and non-professional healthcare workers performing a public health role. This was to contact and provide public health advice to those who test positive for COVID-19, gather their close contacts, inform these of their status as a close contact, and refer them for a test with the aim of breaking the chain of disease [3].

While contact tracing staff are provided with short intensive induction and structured training [2], they operate in a dynamic environment where processes and public health advice are continuously adapted in light of emerging knowledge and evidence related to the pandemic [4]. During surges in COVID-19 cases, staff face pressure to trace high numbers of close contacts of infected cases [6], and given their varied backgrounds, prior training, experience, and coping mechanisms, they may experience difficulties in managing the anxieties and emotional distress of members of the public.

There was a global increase in the prevalence of anxiety and depression disorders in the general public during 2020; locations with increasing COVID-19 infection rates and reduced human mobility were associated with the greatest increases in prevalence [7]. While mental health symptoms were elevated among general populations in the early months of the pandemic, there is evidence of a normalisation of rates toward the end of 2020 and early 2021 both globally [8] and in Ireland [9]. This may represent a broad adaptation among the general public to the pandemic [10], and is in line with the expectation that the majority of people do not develop psychopathologies after natural disasters [11]. However, there is evidence that the psychological response to the pandemic is not homogeneous. Individuals with a history of mental health treatment, loneliness, and lower resilience showed sustained or increased levels of mental health symptoms from March 2020 to April 2021, indicating a heterogenous psychological response to the pandemic that warrants further exploration [12].

Healthcare workers who work with COVID-19 patients are at increased risk of stress, burnout and secondary trauma [13]. Secondary trauma differs from post-traumatic stress in that the trauma develops from providing empathy and compassion to traumatised individuals [14, 15]. In Japan, healthcare workers showed a sustained increase in psychological distress from March 2020 to November 2020 compared to non-healthcare workers [16]. Between May and September 2020, rates of mental distress among healthcare workers in Argentina rose from 40 to 46% [17]. Sustained psychological impact among healthcare workers was not limited to frontline workers. Among diverse staff in a Canadian hospital, emotional exhaustion rose from 41 to 50% from September 2020 to February 2021 [18].

While there has been an increasing recognition of the psychological impact of the pandemic on frontline healthcare staff [19], [20], little attention has been paid to the impact on less visible healthcare staff [21], including contact tracers. Contact tracing staff are frontline responders who interact with COVID-19 patients, family members and other close contacts who may be experiencing high psychological distress [22]. In recognition of this, contact tracers in the US have been trained to provide psychological first aid (PFA), and provide referrals to mental health resources [23]. The role of telephone crisis line workers, who often experience secondary trauma, stress and burnout [24], is similar to that of contact tracers in that although they are physically distant from COVID-19 patients, they are emotionally present and therefore at risk of secondary traumatic stress [25]. During the Ebola epidemic in West Africa, workers on a helpline for people reporting potential Ebola cases often experienced feelings of distress, helplessness, despair and sadness in response to calls [26]. A cohort of COVID-19 contact tracers in Turkey had similarly high rates of anxiety to healthcare workers, and reported higher rates of insomnia [27]. Additionally, burnout and fatigue were common among workers at a telephone based pandemic related psychological support service in China [28]. Taken together, these studies suggest there is a need to assess the psychological impact of the pandemic on contact tracing staff, particularly on the measures of burnout and post traumatic stress disorder (PTSD).

The COVID-19 pandemic has presented an unprecedented public health challenge [29]. Staff are a contact tracing centre key resource, therefore in order to ensure the wellbeing of contact tracing staff and develop appropriate supports, it is important to understand the psychological impact of this role on staff during the COVID-19 pandemic. Furthermore, it is necessary to understand whether the psychological impact varies according to contact tracers’ demographic profile, such as those without a background in healthcare, to properly inform training and support for all contract tracers.

The research questions guiding this study were twofold: (i) what is the psychological impact of contact tracing work on staff during the COVID-19 pandemic response?, and (ii) does the psychological impact of contact tracing on staff vary according to the demographic profile of contact tracers?

## Methods

### Study design and setting

**Fig. 1:**
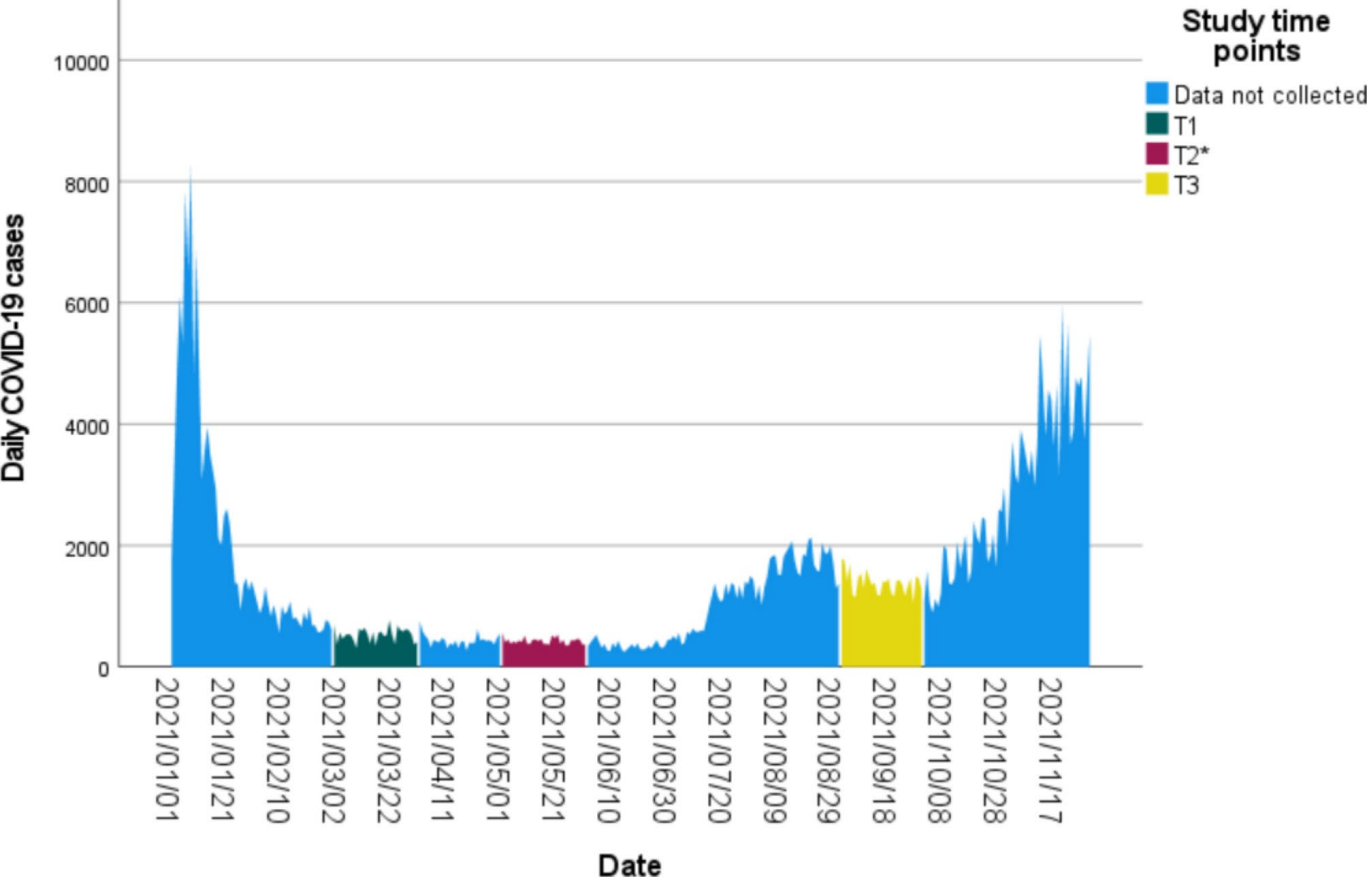
Study timeline *Data from T2 was not included in the analysis due to the low response which occurred as a result of a cyber-attack on the country’s health system

This study used a longitudinal design with three repeated measures. The core inclusion criterion was being employed as a contact tracer in Ireland during the COVID-19 pandemic response. Contact tracers with a professional background in healthcare worked as clinical contact tracers making Call 1s, which entailed informing members of the public that they tested positive for COVID-19. Contact tracers without a background in healthcare worked as non-clinical contact tracers, making Call 2s which were calls to positive patients to collect details of their close contacts [5]. An online survey was disseminated in 2021, the second year of the COVID-19 pandemic, via contact tracing centre team leads to all contact tracing staff working in COVID-19 Contact Management Programme (CMP) CTCs in Ireland in March 2021 using the survey platform Qualtrics (https://www.qualtrics.com). Data were collected over the month of March 2021 (T1). Survey dissemination and data collection was repeated for the month of May 2021 (T2), and again in August 2021 (T3), (see Fig. 1). Data collection was spaced in this way to capture prospective stages of the pandemic. The protocol paper for this study includes further detail regarding the study design [30]. A cyber-attack on the country’s health service [30] impacted the research team’s ability to disseminate the survey in May 2021 and for much of August, resulting in a low sample size for May, and a need to prolong the August data collection into September using hard copy dissemination. As a result, there was a low response rate at May (T2) and therefore the data for this time point were not included in this analysis. Additionally, there was a degree of repeated cross-section for T1 and T3 as not all participants that took part at T1 took part at T3 and vice versa due to both the effect of the cyber-attack and the time constraints of participants. The number of participants included in the final analysis was 338.

### Measures

Data were collected on five variables of interest: exhaustion related burnout, perceived stress, mental distress, tension and pressure, and PTSD symptoms. The two primary outcomes of interest were exhaustion related burnout and PTSD symptoms. For each of the five outcome measures, respondents were asked to complete the scale while thinking of their experience as a contact tracer.

Exhaustion related burnout was measured using the exhaustion subscale from the 16-item *Oldenburg Burnout Inventory (*OLBI) [31]. The exhaustion subscale, consists of eight items, including both positively and negatively worded items. The items are scored on a four-point Likert scale ranging from one (strongly agree) to four (strongly disagree), with summed scores ranging from 8 to 32. Higher scores indicate a higher degree of burnout related exhaustion. The English translation of the OLBI is well validated and considered robust [32]. The values for Cronbach’s alpha were α =·71 at T1, and ·80 at T3, indicating good levels of reliability.

Perceived stress was measured using The *Perceived Stress Scale-14* (PSS) [33]. This is a 14-item measure of perceived stress within the past month. It is scored on a five-point Likert scale ranging from zero (never) to four (very often). Scores for the scale range from zero to 56, with higher scores indicating higher levels of perceived stress. This is a reliable and well validated measure with adequate test-retest reliability [34]. The values for Cronbach’s alpha were α = ·87 at T1, and ·87 at T3, indicating very good levels of reliability.

Mental distress was measured using *General Health Questionnaire 12* (GHQ 12) [35]. This is a reliable and valid, brief screening instrument for recent psychological distress over the past few weeks in relation to work with good internal consistency [36]. It consists of 12 items, six of which are positively worded and six are negatively worded. These are rated on a four-point scale ranging from one (more than usual) to four (much less than usual). Summed scores can range from zero to 36, with higher scores indicating higher levels of psychological distress. The values for Cronbach’s alpha were α =·90 at T1, and ·91 at T3, indicating excellent levels of reliability.

The Tension-Pressure subscale from the *Intrinsic Motivation Inventory* (IMI) [37], was used to assess feelings of tension and being under pressure. It consists of five items, two of which are reverse scored. The items are rated on a seven point scale ranging from one (not at all true) to seven (very true). The summed score ranges from one to seven, with higher scores indicating higher feelings of pressure and tension. The IMI is a widely used measure of intrinsic motivation [38]. The values for Cronbach’s alpha were α =·79 at T1, and ·79 at T3 indicating good levels of reliability.

Post-traumatic stress disorder (PTSD) symptoms were measured using the *Impact of Events Scale-6* (IES-6) [39]. The IES-6 is a brief six item version of the 22-item Impact of Events Scale-Revised [40]. It consists of three subscales covering three symptom clusters of post-traumatic stress disorder: intrusion, hyper-arousal, and avoidance occurring over the past seven days. Scored on a five-point Likert-type scale from one (not at all) to five (extremely), the summed scores can range from 0 to 24, with higher scores indicating a greater post-traumatic stress response. It has been used with healthcare workers [41], and is a reliable and valid screen tool for PTSD symptoms in a variety of populations [39]. The values for Cronbach’s alpha α =·87 at T1, and ·89 at T3, indicating very good levels of reliability.

In addition to the above measures, socio-demographic factors including gender, age, and ethnicity were collected. Other factors captured included length of time involved in contact tracing, number of hours worked per week, location of contact tracing centre, professional background, and whether psychological first aid training (PFA), which is offered to staff, was attended. PFA involved group workshop sessions delivered by a psychologist that focused on the compassionate acknowledgement of others experiences with a focus on interrelated physical, practical and emotional needs being met [42]. Data were also collected on whether respondents were currently receiving treatment for a diagnosed mental health condition to control for possible confounding.

### Statistical analysis

To assess the psychological impact of contact tracing on staff during the COVID-19 pandemic response, two-tailed independent samples *t* tests were conducted to compare mean scores for burnout related exhaustion, perceived stress, mental distress tension and pressure, and PTSD symptoms between the March 2021 (T1) and August-September 2021 (T3) samples. Bonferroni adjustments for multiple comparisons were made (p<·05/5 = 0·01).

Five individual exploratory linear mixed models (LMM) were constructed with restricted maximum likelihood to explore the relationship between exhaustion related burnout, perceived stress, mental distress, PTSD symptoms and tension and pressure with demographic factors over time using data from T1 and T3. Linear mixed models were employed to compensate for missing data and allow for repeated measures on any workers responding at multiple time-points. Pairwise multiple comparisons were performed for significant main effects adjusted for multiple comparisons using Fisher’s Least Significant Difference (LSD). Each model included age, gender, ethnicity, high risk for COVID-19, professional background, length of time contact tracing, and hours worked per week as fixed effects. Attendance of PFA and whether the participant was receiving mental health treatment were included as potential confounding variables.

Subject was included as a random intercept for all five models. Schwarz’s Bayesian Criterion (BIC) was used to select the final model when two interaction terms were considered. Criteria for model selection can be seen in the appendix. F tests and pairwise comparisons were reported. The protocol paper for this study includes detail regarding sample size considerations [30]. Additionally, a multivariate analysis of variance (MANOVA) was used to assess the effect of contact tracing centre location on all five outcome variables. All data were analysed using the SPSS statistical software package version 27.

## Results

The survey was completed by 137 contact tracers at T1 (approx. response rate: 17%), 42 contact tracers at T2 (5%), and 218 at T3 (27%). Due to the low response rate at T2, participants from T2 were not included in the analysis. The follow up rate from T1 to T3 was 12%, with 17 participants responding at each time point. The total number of participants included in the analysis is 338.

The cohorts for T1 and T3 were 67% and 59% female respectively. The largest age category at each time point was 18–29 with 34% of participants at T1 and 56% at T3, followed by the 50–59 age group (25% and 14% respectively), and the 30–39 age group (19% and 12%). At T1, 33% had a professional background in healthcare, while a slightly higher percentage (46%) had a professional background in healthcare at T3. Approximately a third of the sample had attended psychological first aid training at T1 (27%) and T3 (31%). Additional demographic characteristics for participants from T1 and T3 can be found in Table 1.

**Table 1:** Demographic characteristics for participants at T1 and T3

	T1 (N = 137)		T3 (N = 218)	
	No. (%)	Mean (SD)	No. (%)	Mean (SD)
Gender				
Male Female Other	41 (32) 86 (67) 2 (2)		75 (37) 117 (59) 6 (3)	
Age				
18–29 30–39 40–49 50–59 60+ Prefer not to say	44 (34) 25 (19) 18 (14) 32 (25) 6 (5) 4 (5)		110 (56) 24 (12) 15 (8) 27 (14) 14 (7) 8 (4)	
Ethnicity				
White Black Asian Other Prefer not to say	115 (84) 3 (2) 5 (4) 2 (2) 4 (3)		165 (83) 6 (3) 13 (7) 4 (2) 10 (5)	
High risk category for covid-19 (defined by HSE criteria)				
Yes No	15 (12) 114 (88)		25 (13) 171 (87)	
Attended psychological first aid training.				
Yes No	37 (27) 90 (66)		60 (31) 134 (69)	
Professional background				
Healthcare Non-healthcare	42 (33) 87 (67)		73 (46) 87 (54)	
Receiving mental health treatment				
Yes No Prefer not to say	5 (4) 113 (87) 10 (8)		16 (8) 163 (84) 16 (8)	
Contact tracing centre				
Centre 1 Centre 2 Centre 3 Centre 4 Centre 5	39 (32) 15 (12) 26 (21) 40 (29) 1 (0·7)		100 (53) 12 (6) 44 (23) 24 (13) 7 (4)	
Time contact tracing in months		5 (2·5)		7 (3·7)
Hours worked per week		39 (5·9)		39 (6·0)

### The psychological impact on contact tracing staff during the COVID-19 pandemic

To address the first research question, independent samples t tests showed significant worsening in mean scores (M ± SD) from T1 to T3 for burnout related exhaustion (19·47 ± 3·50 vs. 21·32 ± 4·05, p < 0·001), PTSD symptoms (6·91 ± 5·35 vs. 9·19 ± 6·04, p < 0·001) (Table 2) and mental distress (12·64 ± 6·54 vs. 14·69 ± 7·52, p < 0·01). Mean scores for perceived stress (23·24 ± 8·45 vs. 27·19 ± 8·91, p < 0·001) and tension and pressure (3·05 ± 1·21 vs. 3·69 ± 1·35, p < 0·001) also significantly increased over time.

**Table 2:** Changes in mean scores for dependent variables from T1 to T3

		T1 Mean (SD)	T3 Mean (SD)	t value	df	p
Exhaustion related burnout		19·47 (3·50)	21·32 (4·05)	4·50	339	< 0·001
Perceived stress		23·24 (8·45)	27·19 (8·91)	4·10	302	< 0·001
Mental distress		12·64 (6·54)	14·69 (7·52)	2·67	318	0·008
PTSD symptoms		6·91 (5·35)	9·19 (6·04)	3·60	314	< 0·001
Tension and pressure		3·05 (1·21)	3·69 (1·35)	4·54	331	< 0·001

Bonferroni adjustment was applied so that p < 0·01 to be considered statistically significant

### The psychological impact of contact tracing on staff during the COVID-19 pandemic response according to the demographic profile of contact tracers

A MANOVA was run to assess the effect of contact tracing centre location on participant’s mental health. Contact tracing centre location had a significant difference (F_20, 972.72_=2.19, p < .01) Wilk’s Λ = 0.86, partial η^2^ = 0.04 on participant’s overall mental health. However, between subjects effects with Bonferroni adjustment (p < .01 to be considered statistically significant) revealed that contact tracing centre location did not have a significant individual effect on either exhaustion related burnout, perceived stress, tension and pressure, mental distress or PTSD symptom scores. Contact tracing centre location was therefore not included in the mixed model analyses.

An independent LMM was run for each of the five outcome variables. Each LMM included the following variables: age, gender, ethnicity, being in a high risk category for COVID-19, psychological first aid attendance, professional background, currently receiving mental health treatment, length of time working in contact tracing and hours worked per week. Interactions with time were explored for the variables age and professional background.

#### Exhaustion related burnout

Using a linear mixed model, there was an effect of gender (F_2, 227.35_=3.99, p < .05), and of ethnicity (F_4, 225.98_) = 3.15, p < .01), age, (F_5, 210.34_=7.83, p < .001) and the interaction of age with time (F_5, 24.20_ = 6.07, p < .01) on exhaustion related burnout scores. Pairwise comparisons, adjusted for multiple comparisons using Fisher’s LSD, indicated that mean exhaustion related burnout scores significantly increased from T1 to T3 for those aged 18–29 (-1·68, CL^1^, -2·81 to -0·55) p < 0·01), those aged 40–49 (-5·50, CL -7·14 to -3·85, p < 0·001), 50–59 (-1·66, CL -2·96 to -0·37, p < 0·05) and those aged 60+ (-3·70, CL -6·80 to -0·60, p < 0·05) (see appendix B, table B3.).

#### PTSD symptoms (age interaction with time)

While there was no significant effect for the interaction term between age and time for PTSD symptoms, there was a significant effect for age (F_5, 2.10.14_=2.27, p < .05). There was also a significant effect for having received psychological first aid training (F_1, 2.21.45_=7.58, p < .01) on PTSD symptom scores. Pairwise comparisons, adjusted for multiple comparisons using Fisher’s LSD, indicated that mean PTSD symptoms scores for those aged 18–29 increased from T1 to T3 (-2·37, CL -4·56 to -0·19, p < 0·05) (Table B4.).

#### PTSD symptoms (professional background interaction with time)

An interaction term between professional background and time was included in a model for PTSD symptoms, this showed an effect (F_1, 253.98_=6.05, p < .005.), as did having received psychological first aid training (F_1, 2.24_=7.02, p < .01), and age group (F_5, 207.11_, p < .05). Pairwise comparisons, adjusted for multiple comparisons using Fisher’s LSD, indicated that participants with a background in healthcare had low PTSD symptom scores at T1 but showed a significant increase in mean PTSD symptom scores by T3 (-3·90, CL -5·97 to -1·72, p < 0·001) (table B5.), reaching mean scores equivalent to those of participants who did not have a background in healthcare.

#### Perceived stress

For the perceived stress model there was an effect of age (F_5, 193.30_=7.92, p < .001.) on perceived stress scores. Pairwise comparisons, adjusted for multiple comparisons using Fisher’s LSD, showed no significant differences in the change over time across age groups.

#### Tension and pressure

There was no significant effects for tension and pressure. Pairwise comparisons, adjusted for multiple comparisons using Fisher’s Least Significant Difference (LSD), indicated that mean tension and pressure scores for those aged 18–29 increased from T1 to T3 (-0·56, CL -1·09 to -0·47, p < 0·05) (table B8). For those aged 60+, there was trend toward reduced tension and pressure scores by T3 (table B8).

#### Mental distress

While was an effect of age in the mental distress model (F_5, 215.62_=3.91, p < .01), and gender (F_2, 227.10_,p < .01) there was no effect of the age interaction with time (F_5, 225.23_=1.33, p = .025) (table B9).

## Discussion

This longitudinal analysis assessed the psychological impact of contact tracing work on staff during the COVID-19 pandemic. In line with international findings indicating that the psychological impact of the pandemic on healthcare workers appeared to be sustained or increased over time [16–18, 43], there was a significant increase in the prevalence of exhaustion related burnout, PTSD symptoms, perceived stress, mental distress and feelings of tension and pressure among contact tracing staff working in Ireland. While contact tracing shares many similarities with call centre work, aside from the finding that call centre workers in The Philippines experienced low levels of exhaustion related burnout [44], there is little comparative research on this work during the COVID-19 pandemic. In the general labour force in the UK there was a reduction in burnout and worker stress, which may be explained by a majority working remotely [45].

The demographic profile of contact tracing staff affected the extent of this psychological impact with those in the youngest age group [18–29] showing significant increases over time in PTSD symptoms, feelings of tension and pressure, and exhaustion related burnout. Contact tracers aged 40–49, 50–59 and 60 + also showed significant increases in exhaustion related burnout from T1 to T3. There was an interesting though nonsignificant trend where mean scores for contact tracers aged 60 + were lower than those of other age groups for perceived stress, burnout, tension and pressure, mental distress and PTSD symptoms at T1 and at T3. For PTSD symptoms, those aged 60 + showed a nonsignificant decrease in scores from T1 to T3. These findings accord with research indicating that older age may be a protective factor against the development of PTSD symptoms [46], burnout [47], mental distress [17, 43], and perceived stress [48] among healthcare workers during the COVID-19 pandemic.

Professional background affected PTSD symptom scores among contact tracers. At T1, PTSD symptom scores were highest in those without a background in healthcare. By T3, PTSD symptom mean scores remained stable at this high level for these participants, while contact tracers with a healthcare background showed a significant increase, with PTSD symptom scores reaching the equivalent of those of their colleagues without healthcare backgrounds.

This pattern of change may be related to the changing nature of the COVID-19 pandemic. In the two months prior to T1, Ireland had experienced a third wave of COVID-19 with a record rate of COVID-19 case numbers [49] and approximately 3% of the Irish population having received a full vaccination [50]. The contact tracing system was rapidly adapted to cope with a tenfold increase in case numbers over a two week period through systems changes including the merging call 1s and call 2s, whereby contact tracers without a background in healthcare made calls informing patients that they have tested positive for COVID-19 [2]. This may have resulted in their speaking with distressed patients [51, 52], and developing symptoms of secondary traumatic stress. Common risk factors for developing secondary trauma symptoms among healthcare workers during the COVID-19 pandemic were having assisted COVID-19 patients [53], having less work experience, a heavy workload and a lack of training [46]. An additional risk factor particular to working with patients over the phone is that contact tracing staff may feel more helpless when dealing with patients in distress [54], particularly during increases in case numbers.

By T3, national hospitalisation and morbidity rates had decreased [55] and 90% of the Irish population had been fully vaccinated [56]. Contact tracers with backgrounds in healthcare were more likely to have received coping skills training in previous roles, and have experience working with distressed patients. However, the cumulative effect of providing empathetic support to patients over the six month period may have led to their developing symptoms of secondary trauma [14, 15]. Additional factors effecting this finding include the level of exposure to distressed members of the public that contact tracers with backgrounds in healthcare had in comparison to those without this background, in addition to variations among healthcare backgrounds and ensuing variations in levels of training in communication on difficult topics prior to contact tracing [57].

These findings suggest that the heterogeneous nature of the psychological impact of the COVID-19 pandemic extends to contract tracers, providing an important addition to the literature on the psychological impact of the COVID-19 pandemic on less visible healthcare workers [21]. A limitation of the study was the low response rate at T2 due to the cyber-attack on the Irish health service as this meant that data from this time point could not be used in the analysis. The differences in sample composition from T1 to T3 may have had an impact on the increase in the prevalence of exhaustion related burnout, PTSD symptoms, perceived stress, mental distress and feelings of tension and pressure. Mapping the psychological impact of the pandemic response with the changing nature of the pandemic is challenging [58]. T1 took place with the end of the third wave in Ireland and T3 took place a month before the beginning of the fourth wave, therefore the pattern of COVID-19 cases and thus contact tracing calls to be made declined over the study period. The timing of the study was thus a limitation as it did not track the real time impact of either wave. Despite this, these findings suggest that the pandemic had a cumulative psychological impact on contact tracing staff. A strength was in linking the research with the CMP with the aim of supporting the implementation of the findings into practice. Rapid reviews of the study findings were disseminated to the CMP at each of the three time points to aid the CMP in developing appropriate supports for contact tracing staff. These included a need for more equitable access to PFA training.

Approximately a third of participants attended PFA training during the study period (with further training planned and delivered since T3 across CTCs). This can foster resilience [59] and assist in the recovery of trauma-exposed individuals [60]. Future research should explore whether different or more targeted supports are required according to demographic profiles. Contact tracing staff in the youngest age group, and in those without a background in healthcare may be at greater risk of adverse mental health outcomes. Employee assistance programs (EAPs) provide support and enable the development of action plans for healthcare workers under psychological stress including secondary trauma [61]. A further recommendation to the CMP was to clearly communicate the availability of EAP to staff, as those with higher rates of PTSD symptoms may benefit from this [62]. Additionally, the HSE Stress Control Programme may be of benefit in providing stress management skills [63], as may the HSE Values in Action Programme and, more generally, occupational health services [62]. Communication of these services could be introduced during the on boarding of new contact tracing staff. Ongoing qualitative research [64] will offer more nuance and understanding of the experiences of contact tracing staff, and identification of factors that could be contributing to the development of adverse mental health outcomes, and that might help mitigate against these.

The study provides an important, initial insight into the psychological impact of the pandemic on contact tracing staff. Contact tracing staff showed increased rates of PTSD symptoms, exhaustion related burnout, perceived stress, mental distress and tension and pressure. Those aged 18–29 experienced a greater vulnerability to PTSD symptoms, and feelings of tension and pressure, while participants in all age groups but those aged 30–39, showed increased exhaustion related burnout. Though not significant, increased age was related to a lower level of symptoms for each outcome at T1 and T3. As the likelihood of future pandemics increases [65], it is imperative that the psychological health of this vital workforce is supported. These findings highlight the importance of providing interventions to both prevent the development of these psychological issues and to support those affected, and the need to research whether targeted interventions would be of greater benefit for contact tracing staff.

## Electronic supplementary material

Below is the link to the electronic supplementary material.

Supplementary Material 1

## Data Availability

The datasets generated during the current study are not publicly available due to the sensitive nature of the research, but are available from Aoife De Brún (aoife.debrun@ucd.ie ) on reasonable request.

## Footnotes

1 Confidence interval.

## List of Abbreviations

PTSD: Post-traumatic stress disorder
WHO: World Health Organisation
HSE: Health Service Executive
PFA: Psychological first aid
CMP: Contact Management Programme
OLBI: Oldenburg Burnout Inventory
PSS: Perceived Stress Scale
GHQ: General Health Questionnaire
IMI: Intrinsic Motivation Inventory
IES: Impact of Events Scale
LMM: Linear mixed models
LSD: Least Significant Difference
MANOVA: Multivariate analysis of variance
