# Supplementary material for: Exploring the psychological impact of contact tracing work on staff during the COVID-19 pandemic
Source: BMC Health Serv Res. 2023 Jun 8;23:602. doi: 10.1186/s12913-023-09566-6 (PMC10250071; doi:10.1186/s12913-023-09566-6)
Supplement: Supplementary file 1 — Supplementary Material 1 [file 12913_2023_9566_MOESM1_ESM.docx]

# Appendix A

The model for exhaustion related burnout, the model included an interaction term between age and time with a BIC of 1311. For perceived stress the model included an interaction term between age and time. This had a BIC of 1694. The tension and pressure model included an interaction term between age and time resulting in a BIC of 870. Two models were considered for mental distress. The first included an interaction term between age and time resulting in a BIC of 1637, while the second included an interaction term between total time spent contact tracing and time resulting in a BIC of 1665, therefore the first model was chosen as the final model

The first model for PTSD symptoms included an interaction term between age and time and was run with the random command in SPSS syntax as were the previous four models, however due to a low number of participants with a background in healthcare who participated at both time point 1 and 3, the final Hessian matrix was not positive definite. As a result the LLM for PTSD symptoms was run using the repeated command using an unstructured covariance matrix. The first model had an interaction term between age and time resulting in a BIC of 1527. The second model included an interaction term between professional background and time, and had a BIC of 1547. While the first model was chosen as the final model for PTSD symptoms, the second model had findings of interest which were also reported.

## Appendix B

| **Table B3. Post hoc pairwise comparisons for exhaustion related burnout** | | | | |
| --- | --- | --- | --- | --- |
| **Age** | **Mean change in exhaustion related burnout scores from T1 to T2** | **Std. error** | **p-value** | **95% CI** |
| 18-29 | -1.68 | 0.56 | <0.01 | (-2.81, -0.55) |
| 30-39 | -1.25 | 1.04 | 0.23 | (-3.32, 0.81) |
| 40-49 | -5.50 | 0.77 | <0.001 | (-7.14, -3.85) |
| 50-59 | -1.66 | 0.63 | 0.01 | (-2.96, -0.37) |
| 60+ | -3.70 | 1.56 | 0.02 | (-6.68, -0.60) |
| Prefer not to say | 5.58 | 3.68 | 0.13 | (-1.67, 12.84) |

| **Table B4. Post hoc pairwise comparisons for PTSD symptom scores with age interaction** | | | | |
| --- | --- | --- | --- | --- |
| **Age** | **Mean change in PTSD symptom scores from T1 to T2** | **Std. error** | **p-value** | **95% CI** |
| 18-29 | -2.37 | 1.11 | 0.03 | (-4.56, -0.19) |
| 30-39 | -1.68 | 1.88 | 0.37 | (-5.40, 2.02) |
| 40-49 | -2.71 | 2.18 | 0.21 | (-7.01, 1.59) |
| 50-59 | -1.01 | 1.59 | 0.52 | (-4.16, 2.14) |
| 60+ | 1.47 | 3.03 | 0.62 | (--4.44, 7.39) |
| Prefer not to say | 11.58 | 5.56 | 0.03 | (0.62, 22.54) |

| **Table B5. Post hoc pairwise comparisons for PTSD symptom scores with professional background interaction** | | | | |
| --- | --- | --- | --- | --- |
| **Professional background** | **Mean change in PTSD symptom scores from T1 to T2** | **Std. error** | **p-value** | **95% CI** |
| Healthcare | -3.48 | 1.16 | <0.01 | (-5.97, -1.72) |
| Non-healthcare | -0.260 | 0.98 | 0.79 | (-2.19, 1.67) |

| **Table B6. Post hoc pairwise comparisons for perceived stress** | | | | |
| --- | --- | --- | --- | --- |
| **Age** | **Mean change in Perceived stress scores from T1 to T2** | **Std. error** | **p-value** | **95% CI** |
| 18-29 | -2.19 | 1.59 | 0.17 | (-5.34, 0.95) |
| 30-39 | -0.76 | 2.71 | 0.77 | (-6.10, 4.58) |
| 40-49 | -3.04 | 3.09 | 0.32 | (-9.16, 3.07) |
| 50-59 | -3.93 | 2.28 | 0.08 | (-8.43, 0.57) |
| 60+ | -4.07 | 4.37 | 0.35 | (-12.69,4.54) |
| Prefer not to say | 15.39 | 8.13 | 0.06 | (-0.63,31.42) |

**Table B7. Change in mean scores for tension and pressure**

| **Age** | **Time** | **Mean** | **Std. Error** |  |
| --- | --- | --- | --- | --- |
|  |  |  |  | **95% CI** |
| 18-29 | Time point 1 | 3.26 | 0.37 | (2.52, 4.00) |
|  | Time point 3 | 3.83 | 0.35 | (3.12. 4.54) |
| 30-39 | Time point 1 | 3.18 | 0.43 | (2.32, 4.04) |
|  | Time point 3 | 3.82 | 0.47 | (2.88, 4.75) |
| 40-49 | Time point 1 | 3.06 | 0.48 | (2.10, 4.02) |
|  | Time point 3 | 3.79 | 0.51 | (2.77, 4.81) |
| 50-59 | Time point 1 | 3.12 | 0.44 | (2.25, 3.99) |
|  | Time point 3 | 3.45 | 0.45 | (2.55, 4.34) |
| 60+ | Time point 1 | 1.87 | 0.69 | (0.50, 3.24) |
|  | Time point 3 | 2.71 | 0.58 | (1.56, 3.85) |
| Prefer not to say | Time point 1 | 3.90 | 1.01 | (1.91, 5.90) |
|  | Time point 3 | 2.06 | 1.00 | (0.08, 4.05) |

| **Table B8. Post hoc pairwise comparisons for tension and pressure** | | | | |
| --- | --- | --- | --- | --- |
| **Age** | **Mean change in Tension and pressure scores from T1 to T2** | **Std. Error** | **p-value** | **95% CI** |
| 18-29 | -0.56 | 0.26 | 0.03 | (-1.09, -0.04) |
| 30-39 | -0.63 | 0.45 | 0.15 | (-1.52, 0.24) |
| 40-49 | -0.72 | 0.50 | 0.48 | (-1.71, 0.26) |
| 50-59 | -0.32 | 0.36 | 0.37 | (-1.04, 0.39) |
| 60+ | -0.84 | 1.36 | 0.25 | (-2.28, 0.60) |
| Prefer not to say | 1.84 | 1.43 | 0.17 | (-0.84, 0.84) |

| **Table B9. Post hoc pairwise comparisons for mental distress** | | | | |
| --- | --- | --- | --- | --- |
| **Age** | **Mean change in mental distress scores from T1 to T2** | **Std. error** | **p-value** | **95% CI** |
| 18-29 | -0.89 | 1.34 | 0.48 | (-3.57, 1.71) |
| 30-39 | 0.93 | 2.25 | 0.69 | (-3.55, 5.32) |
| 40-49 | -2.21 | 2.46 | 0.39 | (-7.01, 2.76) |
| 50-59 | 0.25 | 1.77 | 0.88 | (-3.28, 3.78) |
| 60+ | -2.18 | 3.64 | 0.55 | (-9.37, 5.01) |
| Prefer not to say | 15.72 | 6.89 | 0.02 | (2.13, 29.30) |
